# Telemedicine Clinics in General and Colorectal Surgery During the COVID-19 Pandemic: Patient-Reported Outcomes

**DOI:** 10.7759/cureus.52441

**Published:** 2024-01-17

**Authors:** Onyekachi E Ekowo, Ahmed Elgabry, Nuno Gouveia, Aftab Khan

**Affiliations:** 1 General and Colorectal Surgery, Darent Valley Hospital, Dartford, GBR; 2 General and Colorectal Surgery, Queen Elizabeth The Queen Mother Hospital, Margate, GBR

**Keywords:** qualitative studies, covid-19 impact on general surgery, outpatients clinic, patient reported outcome measures, telemedicine (tm)

## Abstract

Introduction

Telemedicine was introduced in place of face-to-face clinics during the COVID-19 pandemic to limit exposure and spread of the virus. This was an immediate transformation to surgical practice without prior training. Concerns were raised about whether this fulfilled the patient’s expectations. In this study, we investigated patients’ perspectives and feedback about surgical telemedicine clinics. We also investigated factors that may have influenced patient feedback.

Methods

We undertook a retrospective qualitative study between June and August 2020 at the Darent Valley Hospital, England, United Kingdom. A well-structured 5-point questionnaire was designed to capture patients' experiences with the help of non-medical volunteers. Patients were invited to participate, either online or through direct telephone calls. Ancillary data, such as demographics, previous visits, and the physician’s grade, was also collected. A Mann-Whitney U test was used to compare variables.

Results

A total of 198 patients completed the questionnaire (online = 67, telephone = 133, median age 59 years, IQR 44-79, male: female = 1). A rating from ‘good to excellent’ for ‘overall experience’, ‘opportunity to express concerns’, and ‘doctors consultation’ was given by 90%, 93%, and 89.4%, respectively. About 79.8% felt reassured. Given the option, 63% would prefer face-to-face consultation in the future. Telemedicine clinics led by consultant surgeons had statistically significantly better ratings than junior grades.

Conclusion

This is the first study investigating patients’ experiences of telemedicine in general and colorectal surgery. A high proportion of patients rated a satisfactory experience and felt reassured. The majority of patients would still prefer face-to-face consultations in the future. Based on the results of the current study, we would recommend the integration of telemedicine into future secondary care provision in general and colorectal surgery.

## Introduction

During the first wave of the COVID-19 pandemic, there was a rapid transition in service provision in all fields of medical services in the United Kingdom. In order to limit the spread of the virus, all non-emergency hospital services were either suspended or transformed. One of these transformations was the adaptation of telemedicine clinics for the timely delivery of care while minimizing exposure to protect patients and medical practitioners. This involved either a voice call or a video call between the surgeon and the patient. Despite the uncomplicated feasibility of this technology, telemedicine poses some challenges for both the surgeon and the patients, especially as a first encounter. From a surgeon’s perspective, non-verbal communication provides vital cues that supplement clinical history and examination to compose the overall picture of the presenting complaints for the surgeon. A clinical examination is essential for reaching the correct diagnosis for most surgical complaints. Loss of such information on a telemedicine platform can lead to over-investigation and/or treatment and misdiagnosis in some cases [1]. From a patient’s perspective, various issues can be anticipated, ranging from the inability to describe their problem and difficulty experiencing empathy to the poor overall satisfaction of care provision.

While, there are several publications emphasizing the advantages of telemedicine for service provision in general, there is no published study directly recording patients’ perspectives and feedback about telemedicine in general or colorectal surgery [2]. Therefore, the current study was undertaken to obtain insight into the patient’s experience and their perspective on synchronous telemedicine surgical clinics. The primary aim of the study was to obtain patients’ feedback about their experience of the surgical telemedicine clinics. The secondary aim of the study was to investigate any factors that may have influenced the patient’s feedback.

## Materials and methods

Study design and the study population

The study was designed as a retrospective qualitative study. The study was conducted at the Darent Valley Hospital, located in Kent County, England, United Kingdom. The telemedicine clinic started at the end of March 2020, after the COVID-19 restriction came into place nationally. This involved real-time voice telephone calls to the patients during allocated clinic appointments, assessment of their complaints over the phone, reassurance, and clear communication of the plan for the management of their problems, followed by written documentation and a letter to the patient and their general practitioner. The study period was chosen from June 5 to August 8, 2020, in order to exclude any bias arising from the ‘learning curve’ period of telemedicine clinics for surgical doctors. Adult patients who attended the telemedicine clinic for general and colorectal surgery were invited to take part in the study. Only patients with completed data were included in the final analysis (see below for sample size calculation). Consent was obtained from all the patients taking part in the study. The study was designed in accordance with the Consolidated Criteria for Reporting Qualitative Research (COREQ) [3].

The research team and reflexivity

The telephone interview was conducted by three authors (Onyekachi Ekowo (OE), MBBS, Ahmed Elgabry (AE), MBBS, and Nuno Gouveia (NG), specialist nurse practitioner). They did not take part in the telemedicine clinics. All three interviewers are male and worked as junior members of the surgical teams. OE and AE are senior house officers within three years of their post-graduation. NG is a senior nurse who has worked with the surgical team for the last five years. The interviewers had no prior relationship with the study subjects. At the beginning of each interview, the interviewers introduced themselves by their names and grades and followed a strict protocol for obtaining patients' ratings for the questionnaire.

Sample size calculation

**Figure 1:**
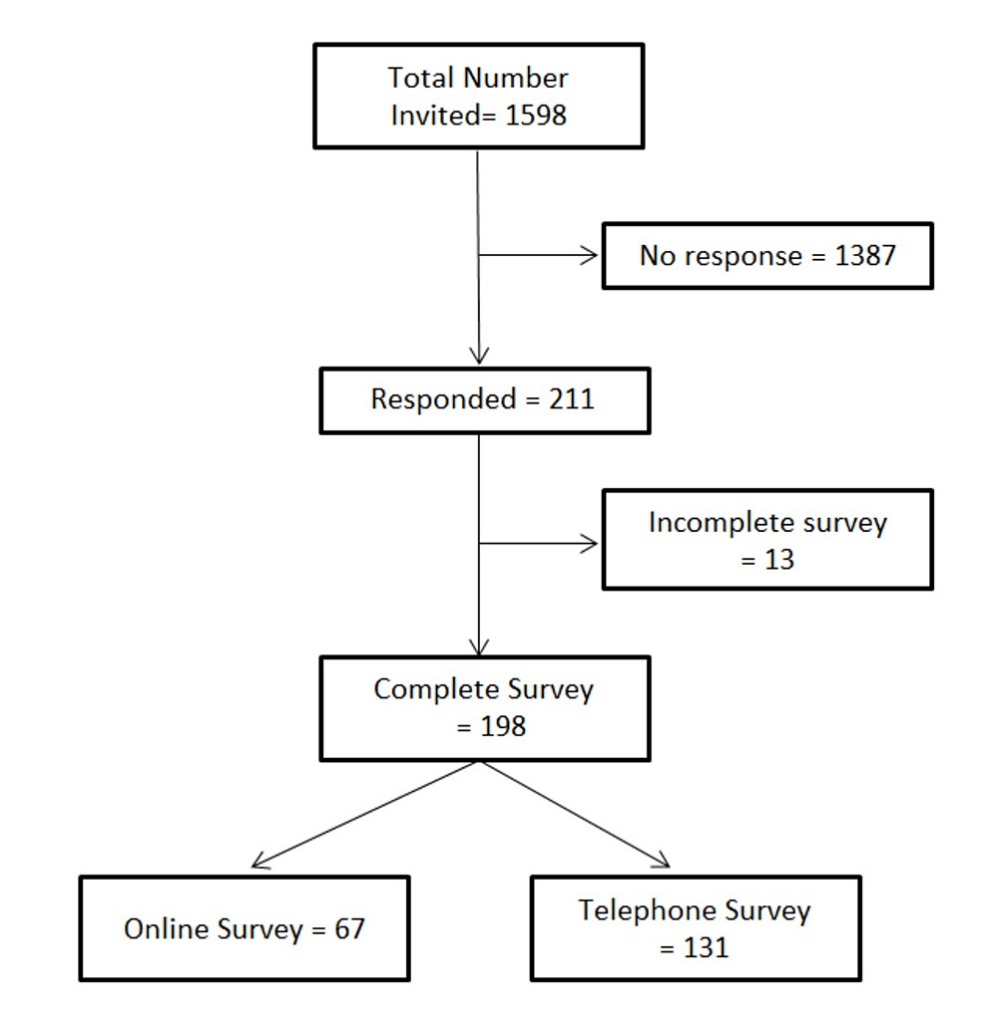
Participant response rates and final sample size The flow chart demonstrates the response and completion rates of the survey. Only completed surveys were included in the final analysis.

Approximately 200 telemedicine appointments per week are allocated between 15 general and colorectal surgical clinics. This would amount to approximately 1598 patients during the study period (from June 5 to August 8, 2021). Only 67 (<5%) patients completed the online survey, and a further 131 (8%) completed the survey over the telephone (Figure 1). Hence, the final sample size of 198 is representative of a total population size of 400 (clinic appointments over two weeks), for a confidence level of 95% that the real value is within ±5% of the surveyed value [4].

Definitions and data collection

A 5-point questionnaire was designed after consultation and feedback from non-medical volunteers (Table 1). The questionnaire was posted on the trust website, accessible through a special link, and all patients who attended the clinic were invited to take part via a generic text message sent to their mobile phone devices. In accordance with the trust information governance policy, the online questionnaire did not ask for demographic information; therefore, other supporting data could not be retrieved for these patients. Patients were also contacted by the interviewers via a direct telephone call. Only patients who did not take part in the online questionnaire were asked to complete the questionnaire over the telephone. In the latter case, the interviewers had access to their demographic information and, hence, supporting data was retrieved from patient electronic records. Supporting data included patient demographics, new or follow-up clinic appointments, and the surgeon’s grade conducting the consultation.

**Table 1:** Questionnaire The 5-point questionnaire was developed with the help of non-medical volunteers

The questionnaire comprised the following questions:
1) How would you rate the telemedicine clinic today (overall the whole experience)?
2) How would you rate the time and opportunity given to you to express your concerns about your condition?
3) How well did the doctor address your medical issues?
4) Did you feel reassured by the telephone consultation?
5) What choice of clinical review would you prefer in the future?

‘New appointment’ included patients who were referred during the pandemic and may not have had any previous experience of attending an outpatient clinic; ‘Follow-up’ included patients who had any hospital encounter before (inpatient stay, outpatient clinic, telemedicine clinic). ‘Previous hospital appointment’ included patients who had face-to-face appointments before the pandemic and, therefore, were familiar with the hospital clinic system. ‘Multiple telemedicine clinics’ included patients who have had more than one telemedicine clinic within the surgical department. 

Statistical analysis

The questionnaire responses are presented as descriptive analyses. The majority of the data obtained was categorical; therefore, continuous data such as age was also converted to a categorical variable (age category < or > 59 years) to maintain similarity in calculations. A Mann-Whitney U test was used to compare qualitative variables. P<0.05 was considered to be statistically significant. Data maintenance and analyses were performed using Microsoft Excel® (2013) and IBM Corp. Released 2019. IBM SPSS Statistics for Windows, Version 26.0. Armonk, NY: IBM Corp.

## Results

A total of 198 patients responded to the questionnaire. The summary of general data is presented in Table 2. The median age of the study group was 59 years (IQR 44-79), and the female-to-male ratio was one. About 23.7% were new referrals, 42.4% were follow-up appointments, 28.3% had previous hospital appointments, and 28.3% had multiple telemedicine clinics. About 11% were seen by surgical consultants and 55% by surgical registrars.

**Table 2:** Summary of general data The table details a summary of demographic data and factors that may influence the responses of the participants

Total	198
Online survey	67
Telephone survey	131
Age Median (IQR)	59 years (44 to 73)
Gender	
Male	60 (30%)
Female	71 (36%)
Visit Type	
New referral	47 (23.7%)
Follow-up appointment	54 (42.4%)
Surgeons Grade	
Consultant	22 (11%)
Registrar	109 (55%)
Previous hospital appointments*	56 (28.3%)
Previous telemedicine clinics**	56 (28.3%)
*prior to COVID-19 pandemic ** after the pandemic started	

**Figure 2:**
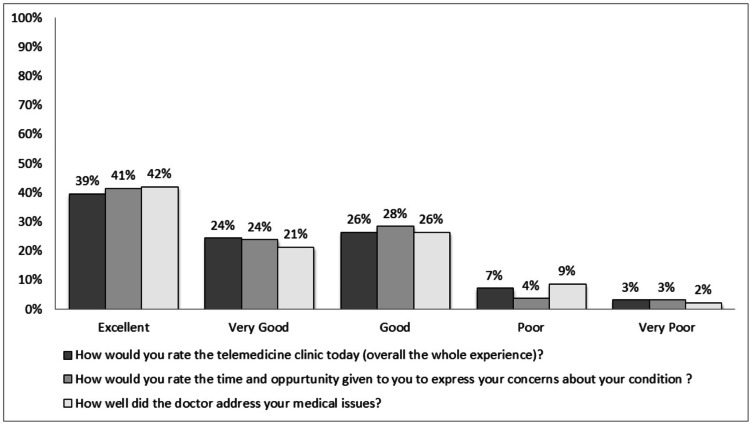
Graphical presentation of responses to questions 1-3

**Figure 3:**
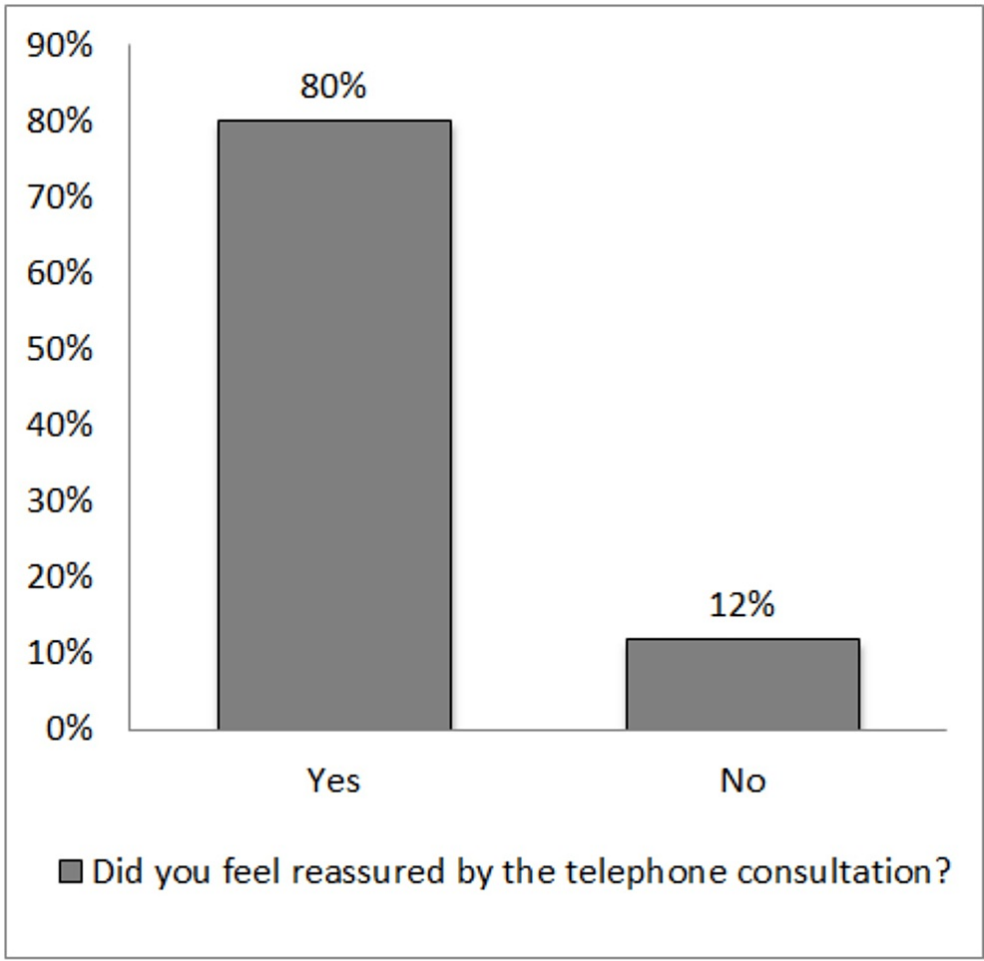
Graphical presentation of the response to question 4

**Figure 4:**
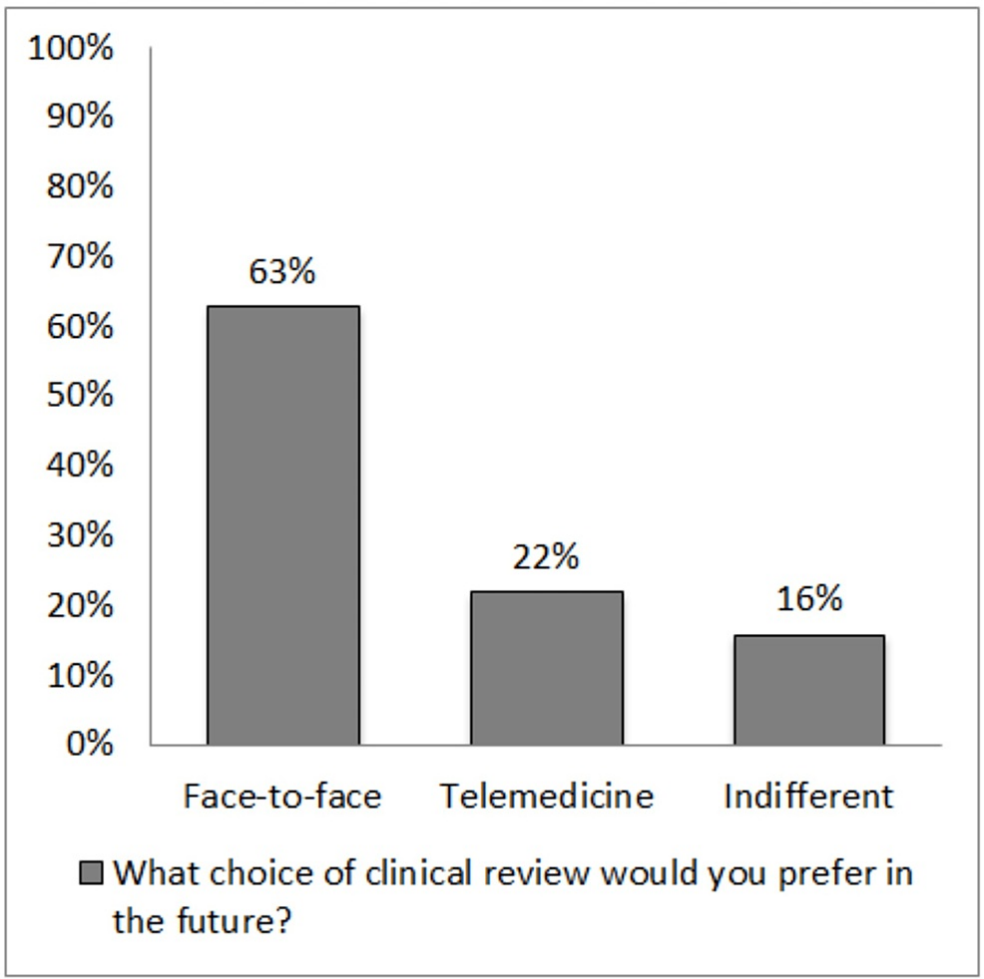
Graphical presentation of the response to question 5

The rating for each question is presented in Table 3 and Figures 2-4. A rating from good to excellent for ‘overall experience’, ‘opportunity to express own concern’, and ‘doctors addressing patient concerns’ was given at 90%, 93%, and 89.4%, respectively. About 79.8% felt reassured, 21.7% would prefer it, and 15.7% felt indifferent about telemedicine for future consultations.

**Table 3:** Descriptive data on responses from the participants The table shows detailed descriptive data on actual responses from the participants

Question 1	How would you rate the telemedicine clinic today (overall experience)?							
	Excellent	Very Good		Good	Poor		Very Poor	p-value
Overall	78 (39.4%)	48 (26.3%)		52 (26.3%)	14 (7.1)		6 (3%)	
Telephone	40 (30.5%)	39 (29.8%)		41 (31.3%)	10 (7.6)		2 (0.8%)	0.02
Online	38 (56.7%)	9 (13.4)		11 (16.4%)	4 (6%)		5 (7.5%)	
Question 2	How would you rate the time and opportunity given to you to express your concerns about your condition?							
	Excellent	Very Good		Good	Poor		Very Poor	p-value
Overall	82 (41.4)	47 (23.7%)		56 (28.3%)	7 (3.5%)		6 (3%)	
Telephone	44 (33.6)	40 (30.5%)		41 (31.3%)	5 (3.8%)		1 (0.8%)	0.08
Online	28 (56.7%)	7 (10.4%)		15 (22.4%)	2 (3%)		5 (7.5%)	
Question 3	How well did the doctor address your medical issues?							
	Excellent	Very Good		Good	Poor		Very Poor	p-value
Overall	83 (41.9%)	42 (21.2%)		52 (26.3%)	17 (8.6%)		4 (2%)	
Telephone	46 (35.1%)	36 (27.5%)		36 (27.5%)	12 (9.2%)		1 (0.8%)	0.13
Online	37 (55.2%)	6 (9%)		16 (23.9%)	5 (7.5%)		3 (4.5%)	
Question 4	Did you feel reassured by the telephone consultation?							
	Yes		No			I don’t Know		p-value
Overall	158 (79.8%)		23 (11.7%)			17 (8.1%)		
Telephone	108 (82.4%)		15 (11.4%)			7 (5.3%)		0.26
Online	50 (75.6%)		8 (11.9%)			9 (13.4%)		
Question 5	What choice of clinical review would you prefer in the future?							
	Fate-to-Face		Telemedicine			Indifferent		p-value
Overall	124 (62.6%)		43 (21.7%)			31 (15.7%)		
Telephone	69 (52.7%)		37 (28.2%)			25 (19.1%)		<0.001
Online	55 (82.1%)		6 (9%)			6 (9%)		

There were statistically significant differences in ratings for online and telephone surveys (Table 3). There were either very high or very low ratings on the qualitative scale and higher preferences for face-to-face clinics.

Amongst the factors that may have influenced the patient’s choice of rating (Table 4 ), telemedicine clinic run by consultant surgeons had statistically significantly better ratings than junior grades.

**Table 4:** The impact of various factors on responses The table presents the statistical analysis of the impact of various factors on the responses of the participants

	Age	Gender	Visit type	Surgeons grade	Previous hospital appointments	Previous telemedicine clinics
	<59 vs >59	Male vs. Female	New vs. Follow-up	Consultant vs. Registrar	Yes vs. no	Yes vs. no
How would you rate the telemedicine clinic today (overall experience)?	0.91	0.95	0.84	0.01	0.87	0.67
How would you rate the time and opportunity given to you to express your concerns about your condition?	0.82	0.86	0.97	0.03	0.43	0.18
How well did the doctor address your medical issues?	0.91	0.98	0.93	0.01	0.84	0.55
Did you feel reassured by the telephone consultation?	0.37	0.54	0.92	0.42	0.84	0.85
What choice of clinical review would you prefer in the future?	0.30	0.89	0.61	0.91	0.49	0.28
Only the p-value is demonstrated in this table. For full data values please refer to supplementary Table 4s						

## Discussion

The analysis shows that the majority of the patients were satisfied with the telemedicine clinic during the COVID-19 pandemic. They were able to express their concerns and also felt satisfied and reassured by the consultation. Patients consulted by consultant surgeons received higher satisfactory ratings on the questionnaire. The patient cohort who completed the online questionnaire rated the telemedicine service at two extremes, either ‘excellent’ or 'very poor’. Despite the high rate of satisfaction with telemedicine, the majority of patients would prefer a face-to-face appointment in the future.

The concept of telemedicine has been developing over the last 20 years; however, its adaptation into clinical practice has been slow [5]. There are many studies supporting the advantages of telemedicine, including cost-effectiveness, improved and timely access to care, and efficiency in follow-up [6]. The COVID-19 pandemic necessitated limiting exposure between individuals to control the spread of the virus, and, therefore, telemedicine was integrated seamlessly into all the United Kingdom National Health Service (NHS) Trusts almost immediately. Although such a transformation helped control exposure for patients and staff alike, concerns remained amongst care providers about whether the same quality of care could be delivered through telemedicine consultations. The current study focused on obtaining feedback from patients about aspects that really concern them, such as time to express their concerns and whether the doctor was able to address their concerns and reassure them. About ≥90% rated ‘good to excellent’ experience (Table 2). A handful of previous studies have evaluated the feasibility and quality of telemedicine from a patient’s perspective. A small qualitative study investigating facilitators and barriers to the use of remote video consultation in patients with musculoskeletal problems highlighted patients' perspectives of increased convenience, fewer waiting times, and better experience in support of telemedicine. On the other hand, difficulty experiencing empathy and reassurance from clinicians, inferior diagnosis and monitoring, privacy, and difficulty staying focused were highlighted as potential barriers [7]. In a larger survey of 399 patients using primary care video consultation, 372 (93.2%) rated virtual visits as ‘high quality’, 379 (95%) felt it was secure and private, and 315 (79%) reported their virtual visit was as thorough as an in-person visit [8]. In a similar study of 152 primary care patients, virtual visits were rated similar to face-to-face consultation on most measures, including time spent with the physician, ease of interaction, and personal aspects of the interaction [9]. It is important to note that all the patients in the aforementioned studies had telemedicine by means of video conference; the majority were based in rural areas (potentially long travel times for face-to-face clinics), and the virtual clinics took place as a follow-up adjunct after an initial face-to-face consultation. In contrast, in the current study, patients had only telephone calls; there was a mixture of new and follow-up appointments, and most patients were local to the study site. There was no statistical difference in response between new and follow-up patients (Table 4). In addition, the current study was conducted in a secondary care setting during a period of pandemic. Despite potential conflicting factors, the responses seen in the current study are similar to previous findings. The overall consensus is that patients rated telemedicine as high quality and felt reassured.

Another interesting finding is that, despite high satisfaction with telemedicine, a vast majority of patients would still prefer a face-to-face consultation (62.6%). Similar findings were reported in a recent study conducted during the COVID-19 pandemic, where 48% of patients using telemedicine clinics for hand surgery would have preferred in-person visits despite the pandemic, and 69% would prefer face-to-face visits after the pandemic [10]. Although not directly asked in the current survey, this may allude to the lack of personal connection and empathy experienced by patients with telemedicine. Such findings have been highlighted in previous studies [7,11]. This may also represent a challenge for telephone-only telemedicine clinics. In addition, none of the doctors had any formal training in conducting telemedicine clinics before its commencement during the pandemic. However, among the factors that influenced patient feedback, consultant-led consultations gained the best ratings. Such findings may be due to the better communication skills that come with experience. One study investigating empathy scores between telemedicine and face-to-face consultation in stroke patients reported no statistical difference, and the authors suggested empathy may be conveyed with advanced communication skills [12]. In a meta-analysis of 635 patients with lung cancer that mostly included studies with telephone follow-up, the telemedicine group reported significantly higher quality of life and lower anxiety and depression scores than the usual care group [13]. This study further highlights the important fact that skilled communication improves the patient experience.

There are some limitations to the study. The retrospective design of the study makes it vulnerable to recall bias. In order to tackle this issue, the questions were designed to obtain an overall view of the experience and avoid any specific details. Although the sample size is only representative of the 400-population size, it is still a large sample size compared to published studies. Only <5% of patients responded to the invitation for the online survey. As the invitation online was completely anonymous, patients were only invited once to avoid duplication of responses. Invited surveys have inherent flaws of selection bias, as only those patients would respond who are conscious of critiquing/improving service. In order to increase compliance, questionnaires were also completed over the telephone. Such an approach may introduce responder bias. Therefore, the questionnaire was designed carefully to omit any leading questions. The interviewers were impartial practitioners and followed a strict protocol, ensuring the patient did not feel pressured toward a particular response. It should be noted that recruitment of a larger sample size and further sophisticated tools for capturing data require considerable resources, which are outside the scope of our unit. Nevertheless, the current study adds valuable patient insight to the current iteration of telemedicine clinics. Despite the lack of formal training for the doctors conducting these clinics, patients rated a high level of satisfaction for most measures.

## Conclusions

In conclusion, the introduction of telemedicine during the COVID-19 pandemic at our unit proved to be an effective transformation. It provided a continued satisfactory level of care provision and reassurance for the patients. Based on the results of the current study, we would recommend the integration of telemedicine into future secondary care provision in general and colorectal surgery.

## Appendices

**Table 5:** Supplemental table Descriptive data of the responses from participants and detailed statistical analysis of the data, including impact on responses of influencing factors

Question 1	How would you rate the telemedicine clinic today (overall experience)?							
Factor	Categories	n	Excellent	Very Good	Good	Poor	Very Poor	p-value
Age	<59	67	22 (32.8)	16 (23.9)	24 (35.8%)	4 (6%)	1 (1.5%)	0.91
	>59	64	18 (28.1%)	23 (35.9%)	17 (26.6%)	6 (9.4%)	0	
Gender	Male	60	19 (31.7%)	18 (30%)	16 (26.7%)	7 (11.7%)	0	0.95
	Female	71	21 (29.6%)	21 (29.6%)	25 (35.2%)	3 (4.2%)	1 (1.4%)	
Visit Type	New	47	19 (40.4%)	7 (14.9%)	15 (31.9%)	6 (12.8%)	6 (12.8%)	0.84
	Follow-up	84	21 (25%)	32 (38.1%)	26 (31%)	4 (4.8%)	1 (1.2%)	
Surgeon’s Grade	Registrar	109	30 (27.5%)	31 (28.4%)	37 (33.9%)	10 (9.2%)	1 (0.9%)	0.01
	Consultant	22	10 (45.5%)	8 (36.4%)	4 (18.2%)	0	0	
Previous Hospital Appointments	Yes	56	13 (23.2%)	24 (42.9%)	17 (30.4%)	1 (1.8%)	1 (1.8%)	0.87
	No	75	27 (36%)	15 (20%)	24 (32%)	9 (12%)	0	
Previous Telemedicine Clinic	Yes	56	17 (30.4%)	18 (32.1%)	18 (32.1%)	2 (3.6%)	1 (1.8%)	0.67
	No	75	23 (30.7%)	21 (28%)	23 (30.7%)	8 (10.7%)	0	

Question 2	How would you rate the time and opportunity given to you to express your concerns about your condition?							
Factor	Categories	n	Excellent	Very Good	Good	Poor	Very Poor	p-value
Age	<59	67	24 (35.8%)	18 (26.9%)	23 (34.3%)	1 (1.5%)	1 (1.5%)	0.82
	>59	64	20 (31.3%)	22 (34.4%)	18 (28.1%)	4 (6.3%)	0	
Gender	Male	60	21 (35%)	17 (28.3%)	20 (33.3%)	2 (3.3%)	0	0.86
	Female	71	23 (32.4%)	23 (32.4%)	21 (29.6%)	3 (4.2%)	1 (1.4%)	
Visit Type	New	47	18 (38.3%)	11 (23.4%)	14 (29.8%)	4 (8.5%)	0	0.97
	Follow-up	84	26 (31%)	29 (34.5%)	27 (32.1%)	1 (1.2%)	1 (1.2%)	
Surgeon’s Grade	Registrar	109	33 (30.3%)	33 (30.3%)	37 (33.9%)	5 (4.6%)	1 (0.9%)	0.03
	Consultant	22	11 (50%)	7 (31.8%)	4 (18.2%)	0	0	
Previous Hospital Appointments	Yes	56	16 (28.6%)	18 (32.1%)	21 (37.5%)	0	1 (1.8%)	0.43
	No	75	28 (37.3%)	22 (29.3%)	20 (26.7%)	5 (6.7%)	0	
Previous Telemedicine Clinic	Yes	56	21 (37.5%)	19 (33.9%)	14 (25%)	1 (1.8%)	1(1.8%)	0.18
	No	75	23 (30.7%)	21 (28%)	27 (36%)	4 (5.3%)	0	

Question 3	How well did the doctor address your medical issues?							
Factor	Categories	n	Excellent	Very Good	Good	Poor	Very Poor	p-value
Age	<59	67	24 (35.8%)	17 (25.4%)	21 (31.3%)	4 (6%)	1 (1.5%)	0.91
	>59	64	22 (34.4%)	19 (29.7%)	15 (23.4%)	8 (12.5%)	0	
Gender	Male	60	22 (36.7%)	15 (25%)	16 (26.7%)	7 (11.7%)	0	0.98
	Female	71	24 (33.8%)	21 (29.6%)	20 (28.2%)	5 (7%)	1 (1.4%)	
Visit Type	New	47	18 (92.3%)	11 (23.4%)	12 (25.5%)	6 (12.8%)	0	0.93
	Follow-up	84	28 (33.3%)	25 (29.8%)	24 (28.6%)	6 (7.1%)	1 (1.2%)	
Surgeon’s Grade	Registrar	109	34 (31.2%)	30 (27.5%)	32 (29.4%)	12 (11%)	1 (0.9%)	0.01
	Consultant	22	12 (54.5%)	6 (27.3%)	4 (18.2%)	0	0	
Previous Hospital Appointments	Yes	56	19 (33.9%)	17 (30.4%)	16 (28.6%)	3 (5.4%)	1 (1.8%)	0.84
	No	75	27 (36%)	19 (25.3%)	20 (26.7%)	9 (12%)	0	
Previous Telemedicine Clinic	Yes	56	21 (37.5%)	15 (26.8%)	16 (28.6%)	3 (5.4%)	1 (1.8%)	0.55
	No	75	25 (33.3%)	21 (28%)	20 (26.7%)	9 (12%)	0	

Question 4	Did you feel reassured by the telephone consultation?					
Factor	Categories	n	Yes	No	I don’t Know	p-value
Age	<59	67	55 (82.1%)	7 (10.4%)	5 (7.5%)	0.37
	>59	64	23 (82.8%)	8 (12.5%)	2 (3.1%)	
Gender	Male	60	48 (80%)	8 (13.3%)	3 (5%)	0.54
	Female	71	60 (84.5%)	7 (9.9%)	4 (5.6%)	
Visit Type	New	47	36 (76.6%)	7 (14.9%)	4 (8.5%)	0.92
	Follow-up	84 (-1)*	72 (86.7%)	8 (9.6%)	3 (3.6%)	
Surgeon’s Grade	Registrar	109	88 (80.7%)	14 (12.8%)	6 (5.5%)	0.42
	Consultant	22	20 (90.9%)	1 (4.5%)	1 (4.5%)	
Previous Hospital Appointments	Yes	56	48 (85.7%)	5 (8.9%)	2 (3.6%)	0.84
	No	75	60 (80%)	10 (13.3%)	5 (6.7%)	
Previous Telemedicine Clinic	Yes	56	50 (89.3%)	5 (8.9%)	1 (1.8%)	0.85
	No	75 (-1)*	58 (77.3%)	10 (13.3%)	6 (8%)	

Question 5	What choice of clinical review would you prefer in the future?					
Factor	Categories	n	Fate-to-Face	Telemedicine	Indifferent	p-value
Age	<59	67	31 (35.8%)	24 (35.8%)	12 (17.9%)	0.30
	>59	64	38 (59.4%)	13 (20.3%)	13 (20.3%)	
Gender	Male	60	33 (55%)	14 (23.3%)	13 (21.7%)	0.89
	Female	71	36 (50.7%)	23 (32.4%)	12 (16.9%)	
Visit Type	New	47	23 (48.9%)	15 (31.9%)	9 (19.1%)	0.61
	Follow-up	84	46 (54.8%)	22 (26.2%)	16 (19%)	
Surgeon’s Grade	Registrar	109	59 (54.1%)	27 (24.8%)	23 (21.1%)	0.91
	Consultant	22	10 (45.5%)	10 (45.5%)	2 (9.1%)	
Previous Hospital Appointments	Yes	56	31 (55.4%)	16 (28.6%)	9 (16.1%)	0.49
	No	75	38 (50.7%)	21 (28%)	16 (21.3%)	
Previous Telemedicine Clinic	Yes	56	33 (58.9%)	6 (9%)	6 (9%)	0.28
	No	75	36 (48%)	24 (32%)	15 (20%)	
n – total number available with completed data * - missing data						
